# Higher *T*_c_ and Upper Critical Field in Novel Misfit Layered Compound Obtained by Indium-Addition Synthesis

**DOI:** 10.3390/ma19132868

**Published:** 2026-07-05

**Authors:** Shogo Kuwahara, Chiaya Yamamoto, Junji Yamanaka, Masanori Nagao, Tadataka Watanabe, Satoshi Demura

**Affiliations:** 1 College of Science and Technology, Nihon University, 1-8-14, Surugadai, Kanda, Chiyoda 101-8308, Tokyo, Japan; watanabe.tadataka@nihon-u.ac.jp (T.W.); demura.satoshi@nihon-u.ac.jp (S.D.); 2 University of Yamanashi Core Facility Center, Yamanashi University, 4-3-11, Takeda, Kofu 400-8511, Yamanashi, Japan; chiayay@yamanashi.ac.jp (C.Y.); jyamanak@yamanashi.ac.jp (J.Y.); 3 Center of Crystal Science and Technology, Yamanashi University, 7-32, Miyamae, Kofu 400-0021, Yamanashi, Japan; mnagao@yamanashi.ac.jp

**Keywords:** single crystal, superconductivity, misfit layered compound, niobium diselenide

## Abstract

Indium-addition synthesis of a misfit layered compound (SnSe)_1.16_(NbSe_2_) was found to obtain a novel sample (In-sample) with another stacking structure in (SnSe)_1.16_(NbSe_2_), causing the increase in the superconducting transition temperature *T*_c_ and the in-plane upper critical field *μ*_0_$H_{c2}^{\mathrm{in}-\mathrm{plane}}$ (0). Crystal structure analysis using single crystals revealed that the In-sample has a significantly elongated lattice constant along the *c* axis due to thickening of the layer other than the NbSe_2_, while retaining the original misfit layered structure. The *T*_c_ increased from 3.6 K to 5.4 K in the In-sample. Furthermore, the in-plane upper critical fields *μ*_0_$H_{c2}^{\mathrm{in}-\mathrm{plane}}$ (0) exceeded the Pauli limit *μ*_0_*H*_p_, reaching 29.4 T (*μ*_0_*H*_p_ ~ 10 T). The coherence length along the *c* axis was reduced in the In-sample, indicating enhanced two-dimensionality. These results suggest that the In-sample not only has another stacking structure but also exhibits higher *T*_c_ and *μ*_0_*H*_c2_.

## 1. Introduction

Superconductivity in a material without spatial inversion symmetry has been extensively investigated due to the appearance of novel properties, such as the topological or parity-mixed superconducting state, the superconducting diode effect, and others [1,2,3]. Although these materials are rare, the range of candidate substances has been expanded to include materials without local spatial inversion symmetry [4]. One of the candidates is a misfit layered compound. Misfit layered compounds (*MX*)*_m_*(*TX*)*_n_* (*m*, *n* = layer numbers) have a unique crystal structure: insulating monochalcogenide *MX* layers (*M* = Sn, Pb and Bi) and conducting dichalcogenide *TX*_2_ layers (*T*: transition metal, *X* = S, Se and Te) are alternately stacked through Van der Waals forces. The spatial inversion symmetry is broken locally at the boundary between the *MX* layer and the *TX*_2_ layer because these layers have different lattices: *MX* and *TX*_2_ layers have a NaCl-type square lattice and a triangular prism lattice [5,6,7,8,9]. Misfit layered compounds with metallic properties often show superconductivity [10,11,12,13,14,15]. The superconducting temperature *T*_c_ value varies by the number of *MX* and *TX*_2_ layers [16]. Furthermore, this unique crystal structure gives rise to distinctive properties, such as a quite high upper critical magnetic field (*μ*_0_*H*_c2_). Here, we focus on NbSe_2_-based misfit layered compounds (*M*Se)*_m_*(NbSe_2_)*_n_* (*M* = Sn, Bi, Pb, La).

The (*M*Se)*_m_*(NbSe_2_)*_n_* system features a structure in which superconducting 2*H*-NbSe_2_ layers are alternately stacked with insulating *M*Se layers [17]. A *T*_c_ of (SnSe)*_m_*(NbSe_2_)*_n_* is approximately 4 K for (*m*, *n*) = (1, 1), and approximately 5 K for (*m*, *n*) = (1, 2). The in-plane upper critical field (*μ*_0_$H_{c2}^{\mathrm{in}-\mathrm{plane}}$) reaches the Pauli limit (*μ*_0_*H*_p_) when (*m*, *n)* = (1, 1) but increases to approximately 1.5 times *μ*_0_*H*_p_ when (*m*, *n*) = (1, 2) [18,19]. In the case of (LaSe)*_m_*(NbSe_2_)_n_, the *μ*_0_$H_{c2}^{\mathrm{in}-\mathrm{plane}}$ reaches nearly 10 times *μ*_0_*H*_p_ for (*m*, *n*) = (1, 1), and approximately twice *μ*_0_*H*_p_ for (*m*, *n*) = (1, 2) [20]. (PbSe)*_m_*(NbSe_2_)*_n_* for (*m*, *n*) = (1, 1) also shows a higher *μ*_0_$H_{c2}^{\mathrm{in}-\mathrm{plane}}$ exceeding *μ*_0_*H*_p_ [21]. On the other hand, (BiSe)_1.10_(NbSe_2_) does not have a higher *μ*_0_$H_{c2}^{\mathrm{in}-\mathrm{plane}}$ but shows an anisotropic superconducting property with two-fold symmetry in the in-plane: the upper critical magnetic field along the *b* axis (*μ*_0_$H_{c2}^{\left|\right|b}$) is about twice as large as that along the *a* axis (*μ*_0_$H_{c2}^{\left|\right|a}$) [22,23]. These novel superconducting states appear in the conducting NbSe_2_ layer. However, the bulk NbSe_2_ does not show these novel properties: the NbSe_2_ single crystal has a *T*_c_ of about 7.2 K, which is higher than that of the NbSe_2_-based misfit layered compound. The *μ*_0_*H*_c2_(0) along the inter-plane and the in-plane of NbSe_2_ are 4.1 T and 12.3 T (0.9 *μ*_0_*H*_p_) [24]. These results suggest that the insertion of the *MX* layer between the NbSe_2_ layers enhances *μ*_0_*H*_c2_(0) and decreases *T*_c_. However, the mechanism for the change in these superconducting properties in the misfit layered compound has not been revealed so far. An elucidation of the mechanism is essential because the novel superconducting state in the misfit compound can be controlled.

In this study, we demonstrated that Indium (In)-addition synthesis stabilized a novel sample with a different stacking structure (In-sample) compared with (SnSe)_1.16_(NbSe_2_), which exhibits an enhanced superconducting state. Single crystals of (SnSe)_1.16_(NbSe_2_) with or without Indium addition were successfully obtained by the molten salt flux method. The lattice constant along the *c* axis of the In-sample increased in comparison with (SnSe)_1.16_(NbSe_2_), which originates from the layer other than NbSe_2_ thickening, while maintaining the misfit stacking structure. The *T*_c_ increased from 3.6 K to 5.6 K in the In-sample. The $H_{c2}^{\left|\right|a}$/*H*_p_ (0) and $H_{c2}^{\left|\right|b}$/*H*_p_ (0) increased twice in value compared to (SnSe)_1.16_(NbSe_2_). These results indicate that the In-sample exhibits an enhanced superconducting state relative to (SnSe)_1.16_(NbSe_2_).

## 2. Experimental/Methods

Single crystal samples were synthesized using the molten salt flux method. In the molten salt flux method, the target single crystals are obtained together with byproducts. Therefore, after synthesis, the target crystals were picked up from the obtained products. Since the composition of the obtained crystals often differs from the nominal starting composition, compositional analysis is necessary to determine the actual composition. We prepared two types of samples, with or without the addition of In. A sample without In addition (denoted as the ref. sample) was prepared to weigh to a stoichiometric ratio of Se: Nb: Sn = 5.16: 2: 1.16 mol% to a total of 0.8 g. The other sample with the In addition (denoted as the In-sample) was also prepared with a ratio of Se: Nb: In: Sn = 5.16: 2: 0.8: 0.2 mol% to a total of 0.8 g. The purities of the reagents are as follows: Se (99.9%; Kojundo Chemical Laboratory Co., Ltd., Sakado, Saitama, Japan), Nb (99.9%; Kojundo Chemical Laboratory Co., Ltd., Sakado, Saitama, Japan), In (99.99%; Soekawa Rikagaku Co., Ltd., Itabashi, Tokyo, Japan) and Sn (99.99%; Kojundo Chemical Laboratory Co., Ltd., Sakado, Saitama, Japan). A mixture of KCl (99.9%; Kojundo Chemical Laboratory Co., Ltd., Sakado, Saitama, Japan) and CsCl (99.9%; Iwatani Corporation, Osaka-shi, Osaka, Japan) was used as the flux, weighed at a 3: 5 mol% ratio to a total of 5 g. The metallic elements were first mixed in an agate mortar, followed by the addition of KCl and CsCl, and thoroughly mixed again. The mixture was then vacuum sealed in a quartz tube and sintered in an electric furnace. The thermal process is as follows: the temperature is increased to 700 °C, followed by slow cooling to 600 °C at a rate of *v* (°C/h). The rate of *v* is 0.5 for the ref. sample and 1.0 for the In-sample. After sintering, the mixture in the quartz tube was taken out and immersed in pure water to remove the flux. The obtained single crystals were characterized by X-ray diffraction (XRD; Ultima IV, Rigaku, Akishima, Tokyo, Japan), Scanning Electron Microscopy (SEM; S-2150, Hitachi High-Tech Corporation, Minato, Tokyo, Japan) with Energy Dispersive X-ray Spectroscopy (EDX; S-775X1, HORIBA, Ltd., Minami, Kyoto, Japan) for compositional analysis, and Transmission Electron Microscopy (TEM; Tecnai Osiris, Thermo Fisher Scientific, Hillsboro, OR, USA) measurements for structural confirmation. The XRD measurements were performed using Cu Kα radiation over the 2*θ* range of 10–70°. For compositional analysis, EDX spectra were measured on freshly cleaved crystal surfaces. The measurement was carried out over several regions of approximately 50 × 50 μm^2^, and five different spots were measured in each region. A composition was determined from the average of these measurements. In addition, some samples were measured by synchrotron X-ray diffraction at BL02B1 in SPring-8 (2024B1917). Superconducting properties were evaluated using PPMS (PPMS; Quantum Design, Inc., San Diego, CA, USA) and MPMS (MPMS; Quantum Design, Inc., San Diego, CA, USA). The current was applied along the a axis for electrical resistivity measurements. In addition, the magnetic fields were applied along the *a*, *b*, and *c* axes when the upper critical field was evaluated. A previous report on (BiSe)_1.10_(NbSe_2_) showed the streak on the crystal surface along the *b* axis [23]. An analogous streak was observed on the surface of both the ref. and In-samples. We therefore identified the direction parallel to the streak as the *b* axis and another direction perpendicular to it as the *a* axis. For the magnetization measurements, a thin platelet-like single crystal with approximate dimensions of 1 mm × 1 mm × 10 μm was used. The magnetic field was applied perpendicular to the platelet plane, namely along the *c* axis.

## 3. Results and Discussions

**Figure 1:**
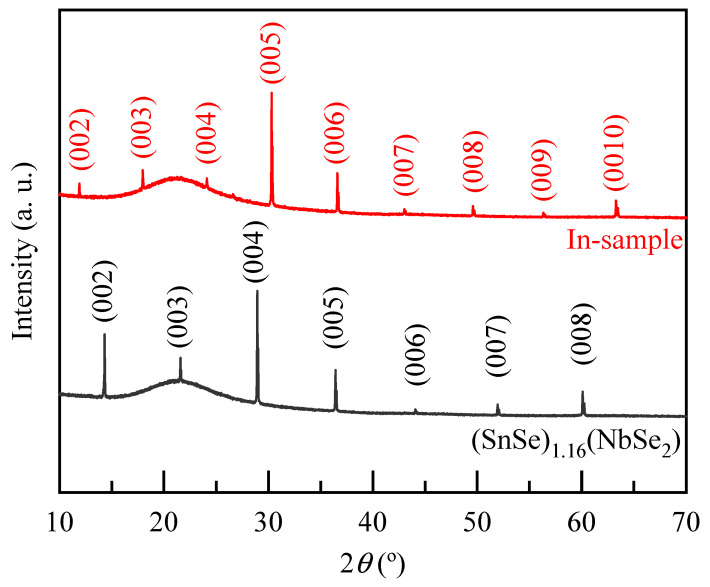
XRD patterns reflected (00*l*) of the ref. sample (SnSe)_1.16_(NbSe_2_) and the In-sample.

XRD results for each sample are shown in Figure 1. The peaks corresponding to the (00*l*) planes were observed because the X-ray was irradiated onto the *ab*-plane of the single crystals. The period of the (00*l*) planes was estimated to be 12.32(2) Å for the ref. sample and 14.73(4) Å for the In-sample. This result indicates that the interlayer distance along the *c* axis is expanded in the In-sample.

**Figure 2:**
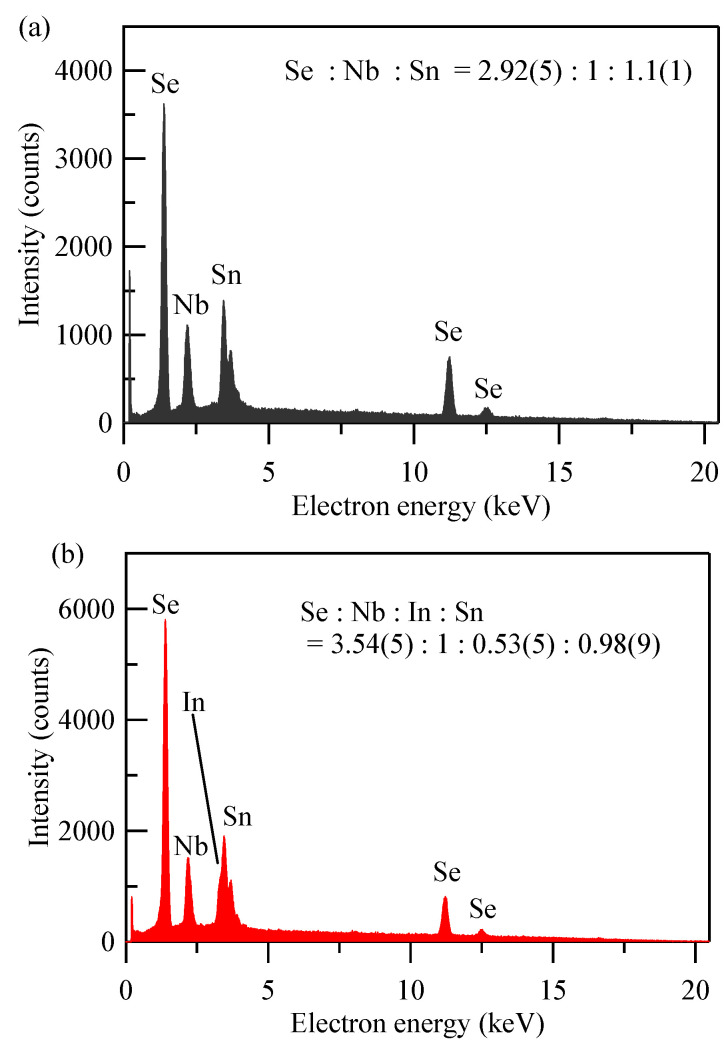
Spectra of EDX measurements of each sample: (**a**) ref. sample and (**b**) In-sample.

Compositional analysis results using SEM-EDX measurements are depicted in Figure 2. To examine the crystal homogeneity, SEM-EDX measurements were performed at multiple points on the sample surface. Composition ratios were calculated from this multi-point analysis. The atomic ratio for the ref. sample is Se: Nb: Sn = 2.92(5): 1: 1.1(1), while that for the In-sample is Se: Nb: In: Sn = 3.54(5): 1: 0.53(5): 0.98(9). These results indicate that single crystals of (SnSe)_1.16_(NbSe_2_) and the In-sample with the above composition were obtained under the present synthesis conditions. However, this compositional change cannot be explained by a simple intercalation or a partial substitution of In for Sn in the ref. sample, as discussed later. Furthermore, the composition is independent of the measurement locations, indicating that a crystal with little inhomogeneity is obtained.

**Figure 3:**
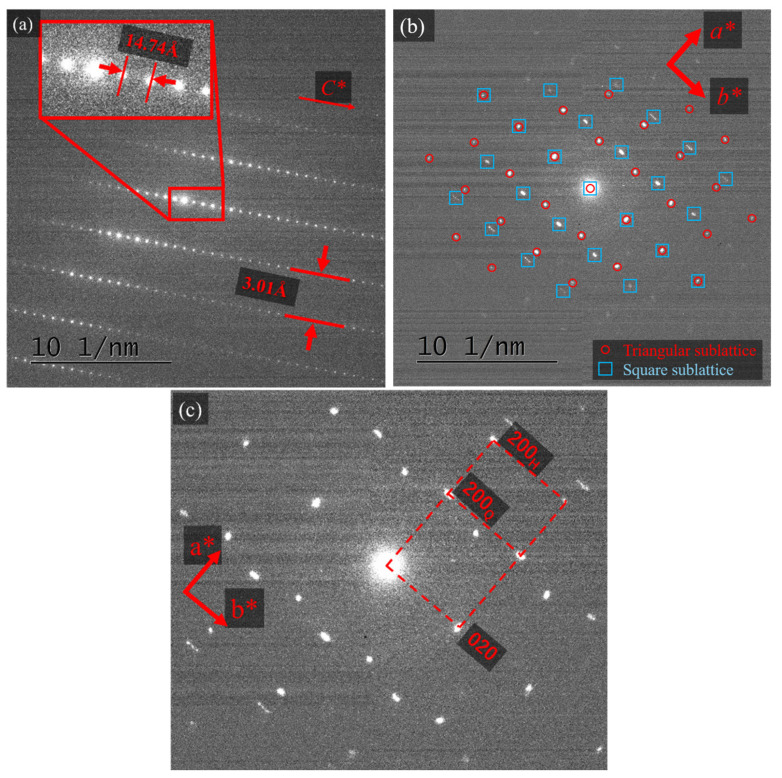
Selected area electron diffraction (SAED) patterns of the In-sample obtained via TEM measurements: (**a**) a*c*-plane, (**b**) a*b*-plane and (**c**) magnified figure of (**b**).

Crystal structure analysis was performed using TEM and synchrotron XRD measurements of the In-sample to obtain more information about the crystal structure. SAED patterns obtained by TEM measurements are depicted in Figure 3. Figure 3a shows the diffraction patterns of the a*c*-plane. The stacking period along the *c* axis is approximately 14.77 Å, consistent with the XRD results. The diffraction pattern of the a*b*-plane in Figure 3b shows distinct patterns composed of triangular lattice spots (red circles) and square lattice spots (blue squares). These patterns are analogous to a previous TEM study of a misfit compound (SbS)_1+*δ*_(NbS_2_) [25]. Its analogous patterns indicate that the In-sample remains a misfit stacking structure consisting of a layer with a triangular lattice and a square lattice. Lattice constants of the In-sample are calculated from Figure 3 and summarized in Table 1. The lattice constants along the *a* and *b* axes are close to those of previous results [18,19]. The lattice mismatch factor *δ* = 2 ∙ ($a_{{\mathrm{NbSe}}_{2}}$/$a_{\mathrm{SnSe}}$) – 1 is 0.17, which is an analogous value of 0.16 in the ref. sample.

**Figure 4:**
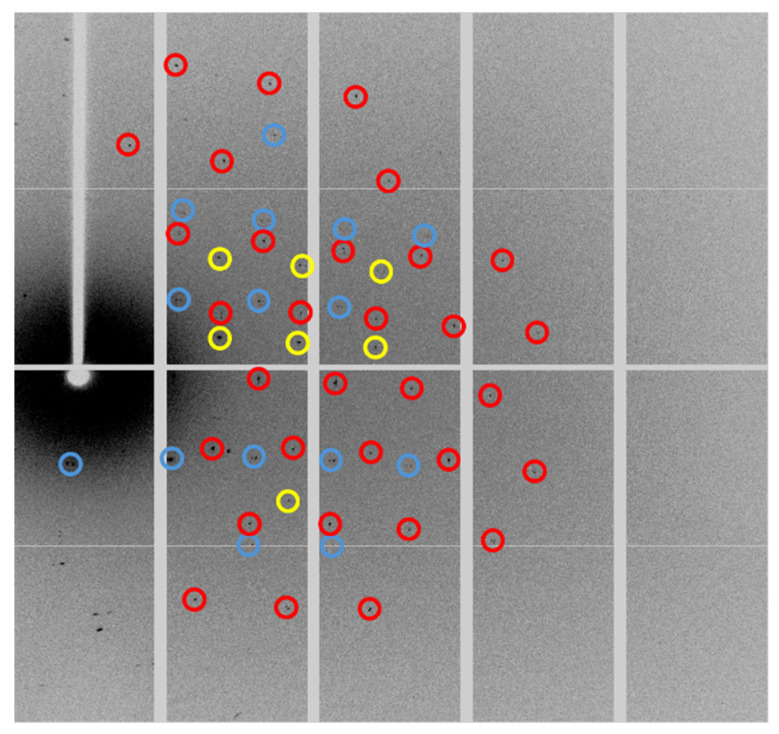
Synchrotron X-ray diffraction images of the (*hk*0) plane for the In-sample single crystals. Diffraction spots originating from NbSe_2_ are marked with red circles, and those from the other layers are marked with blue and yellow circles.

The crystal structure of the In-sample was also evaluated by the synchrotron XRD measurement at Spring-8. Figure 4 shows representative X-ray diffraction images. Diffraction spots attributed to the triangular and square lattices are clearly identified. The lattice constants obtained from these X-ray diffraction patterns are summarized in Table 1. These values are close to those obtained from the SAED patterns. These findings demonstrate that the In-sample not only keeps the misfit stacking structure but also has an enlarged lattice constant along the *c* axis. It is noted that the spots highlighted in yellow in Figure 4 could not be observed in the TEM images. These spots correspond to the reflection from a diagonal direction of the square lattice.

**Figure 5:**
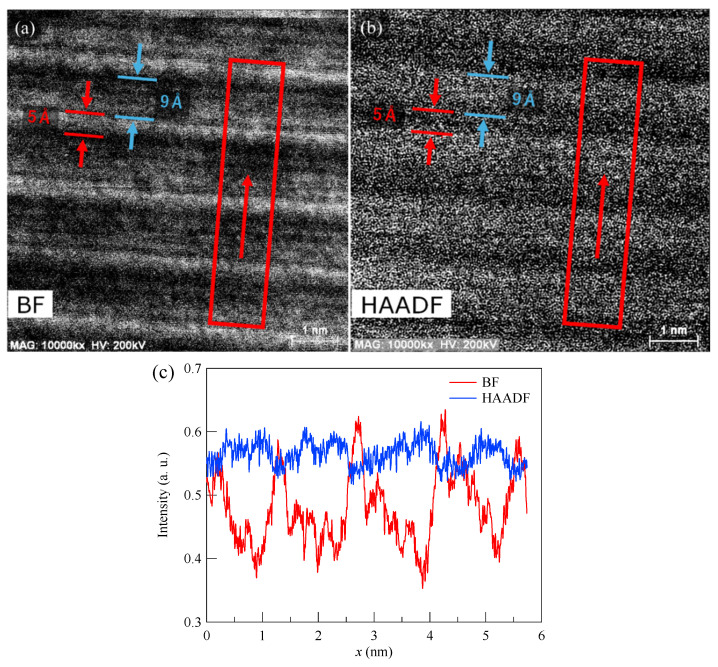
(**a**) Bright-Field (BF) out-of-plane image of the In-sample, and (**b**) High-Angle Annular Dark-Field (HAADF) image. (**c**) Intensity profiles obtained from line-cut analyses along the stacking direction in the BF image (**a**) and the HAADF image (**b**). The regions and directions used for the line-profile analyses are indicated by red squares and arrows in (**a**,**b**), respectively.

In order to determine which of the triangular and square lattice layers is thicker, Bright-Field (BF) and High-Angle Annular Dark-Field (HAADF) images were obtained, as shown in Figure 5. Both images indicate that two layers with different thicknesses are stacked on top of each other. The thickness of each layer is estimated to be approximately 5 Å and 9 Å. The 5 Å layer is brighter in the BF image but darker in the HAADF image, indicating that it consists of lighter elements such as Nb and Se. On the other hand, the 9 Å layer is composed of heavier elements such as Sn and In, as indicated by the same comparison of images. This contrast difference between layers is also confirmed by the line-cut intensity profiles shown in Figure 5c. This BF (HAADF) image and the lattice constants of the triangular lattice in Table 1 indicate that the 5 Å layer is the NbSe_2_ layer. A previous report supports this result: both the SnSe and NbSe_2_ layers have a thickness of approximately 6 Å [26]. On the other hand, the 9 Å layer is not determined to be the SnSe layer, although the lattice constants along the *a* and *b* axes are analogous to those of (SnSe)_1.16_(NbSe_2_) [26]. The type of the 9 Å layer is discussed later. These results suggest that the enlarged layer in the In-sample is not the NbSe_2_ layer but rather the other layer (9 Å layer), resulting in an increased *c* axis lattice constant.

**Figure 6:**
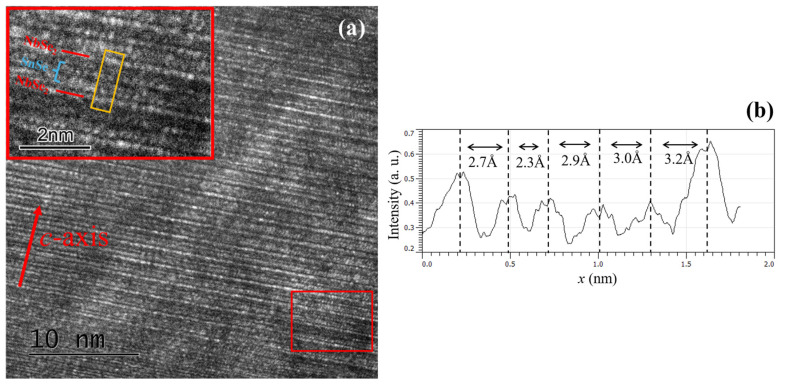
(**a**) High-resolution out-of-plane image of the In-sample single crystal. The inset figure is an enlarged view of the red square. (**b**) Line profile corresponding to the orange square highlighted in (**a**).

Figure 6a shows a high-resolution (HR) image of the *ac*-plane of the In-sample. Brighter stripes are observed along the *c* axis. A line profile along the *c* axis is shown in Figure 6b, which corresponds to the orange square in the inset of Figure 6a. Based on a previous TEM measurement of (SnSe)_1.16_(NbSe_2_), the Nb atoms in the NbSe_2_ layers are brighter than the other atoms in the HR image [27]. Thus, two peaks, located on both sides and of higher intensity, are attributed to Nb atoms in the NbSe_2_ layer. The distance of these peaks is approximately 14 Å, which is consistent with the TEM, XRD and SC-XRD results. Furthermore, the four intermediate peaks between the Nb-related peaks are considered to originate from the structure of the 9 Å layers.

Here, we discuss the crystal structure of the In-sample. The In-sample has an enlarged lattice constant along the *c* axis compared with the ref. sample, which is attributed to the expansion of the 9 Å layers consisting of Sn, In and Se ions. The possibilities for the origins of the expansion are as follows: (1) a substitution of In for Sn in SnSe layers or (2) an intercalation of In between layers in the ref. sample. However, this is not the case, as shown below. (1, 2-1) The composition of Se/Nb deviates from three, which should remain approximately three when the In ion is substituted or intercalated into the ref. sample. (1, 2-2) The lattice expansion is too large to be explained by intercalation. (1, 2-3) The TEM measurements and SAED patterns revealed no intercalated atoms or layers. These results indicate that the In-samples are neither the In-substituted nor the In-intercalated ref. sample (SnSe)_1.16_(NbSe_2_).

The case (2)′, where (InSe_2_) layers are inserted into (SnSe)_1.16_(NbSe_2_), i.e., (SnSe)_1.16_(NbSe_2_)(InSe_2_)*_x_*, is not considered as follows [28,29]. (2′-1) The (SnSe)_1.16_(NbSe_2_)(InSe_2_)*_x_* should have three types of layers in an alternating stacking structure. However, in the out-of-plane TEM images and in the BF and HAADF images obtained in this study, only two types of stacked layers were observed. Furthermore, the line-cut profiles of the TEM images showed four peaks between the NbSe_2_ layers, which cannot be explained by a simple stacking of SnSe and InSe_2_ layers, as in the previous study [28]. (2′-2) The electron diffraction patterns taken with the incident beam perpendicular to the layers showed that only the spots originating from the NbSe_2_ and the square lattice layers were present. If InSe_2_ layers were present, additional diffraction spots corresponding to InSe_2_ should appear, but such spots are not detected. (2′-3) The composition analysis also does not support the insertion of InSe_2_ layers. The EDX composition of the In-sample is Se: Nb: In: Sn = 3.54(5): 1: 0.53(5): 0.98(9). The ratio of Se to In ion becomes Se: In = 0.56: 0.53, where the contribution from SnSe and NbSe_2_ layers (Se: 0.98 + 2.00 = 2.98) is subtracted in the Se composition. This ratio is not consistent with InSe_2_. For this reason, the composition analysis also does not support the formation of additional InSe_2_ layers. From the considerations of (2′-1, 2, 3), we concluded that the insertion of discrete InSe_2_ layers is unlikely.

Another scenario is (3) the insertion of two SnSe layers, where In is partially substituted for Sn, between the NbSe_2_ layers. Based on the above discussion of the square lattice layer, it is highly likely that the thick layer corresponds to two {(Sn,In)Se} layers. Previous reports have shown that the thickness of a single SnSe layer was approximately 6 Å. Therefore, a simple double-SnSe-layer model would give a total thickness of about 12 Å, which is larger than the experimentally observed value of 9 Å. However, partial substitution of In for Sn may reduce the layer thickness because the ionic radius of In^3+^ is smaller than that of Sn^2+^. In fact, the presence of In is confirmed by both EDX and WDS measurements. These results support the interpretation that a partially In-substituted double {(Sn,In)Se} layer is present in our compound. By subtracting the NbSe_2_ component from the EDX composition of the In-sample (Se: Nb: In: Sn = 3.54: 1: 0.53: 0.98), we obtained Se: (In + Sn) = 1.54: 1.51. This composition is comparable to {(Sn, In)Se}_1.5_(NbSe_2_), indicating that the stacking structure consists of two (Sn, In)Se layers and one NbSe_2_ layer. In this case, the expansion of SnSe layers in the In-sample is well explained. This structure aligns with observations from BF, HAADF, and SAED images; it consists of a thick SnSe layer and thin NbSe_2_ layers. Therefore, we finally suggest that the In-sample is (SnSe)_2_(NbSe_2_), where the In ion partially substitutes for the Sn site. On the other hand, an ideal value of 1 + *δ* is 1.16 estimated from the misfit layered compounds (SnSe)_1.16_(NbSe_2_). Thus, the composition with two insulating layers is expected to be {(Sn, In)Se}_2.32_(NbSe_2_). This result suggests that the defect is present in either the (In, Sn)Se or NbSe_2_ layer. Therefore, further investigation of the crystal structure of the defects and related aspects is necessary to determine the detailed structure.

Next, we will compare the main experimental results between the In-sample and ref. sample of (SnSe)_1.16_(NbSe_2_). This comparison is not intended to evaluate a simple doping effect, because the In-sample most likely has a different stacking structure and composition from the ref. sample. As discussed above, the In-sample is most likely an In-substituted (SnSe)_2_(NbSe_2_). The reason for this comparison is that the superconducting properties, such as the upper critical fields, have not been studied in (SnSe)_2_(NbSe_2_), although superconductivity with *T*_c_ ~ 1.9 K has been reported for (SnSe)_2_(NbSe_2_) [27]. (SnSe)_1.16_(NbSe_2_) was therefore chosen as a reference to compare with the In-sample, in which the superconducting NbSe_2_ layer is effectively a single layer in the unit cell, allowing us to discuss these superconducting properties quantitatively.

**Figure 7:**
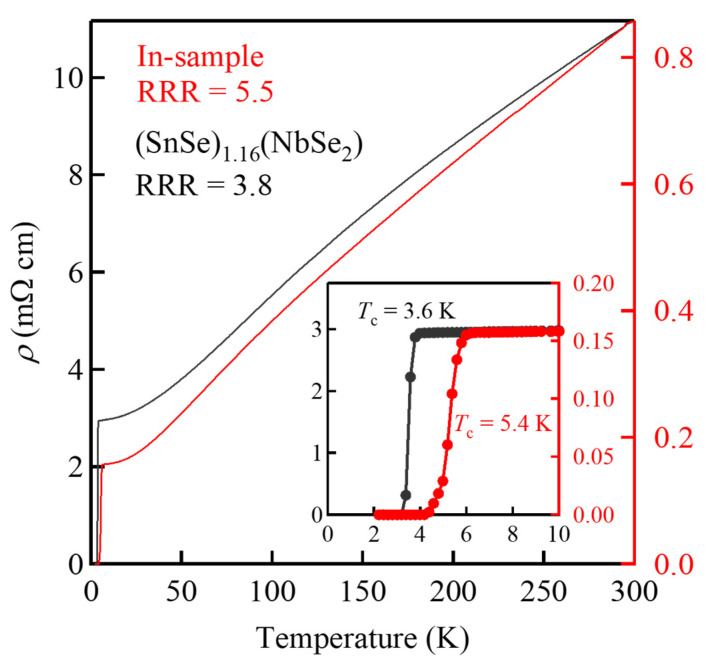
Temperature dependence of electrical resistivity for the ref. and In-samples. The inset shows an enlarged view near the superconducting transition.

Figure 7 shows the temperature dependence of the resistivity measurements for the ref. and In-samples. The current is applied along the *a* axis. Metallic behavior and superconducting transition are observed in both samples. The resistivity of the In-sample is lower than that of the ref. sample by more than one order of magnitude for the entire temperature range. This decrease in resistivity may be attributed to a change in carrier density induced by In-substitution. Furthermore, the higher residual resistivity ratio (RRR) of the In-sample may be attributed to improved crystallinity, although the underlying mechanism remains unclear.

The *T*_c_ is defined as the temperature at which the electrical resistivity decreases to 50% of its normal state value. The *T*_c_ is 3.6 K for the ref. samples, which is consistent with the previous report [18]. On the other hand, the *T*_c_ of the In-sample increases to 5.4 K.

**Figure 8:**
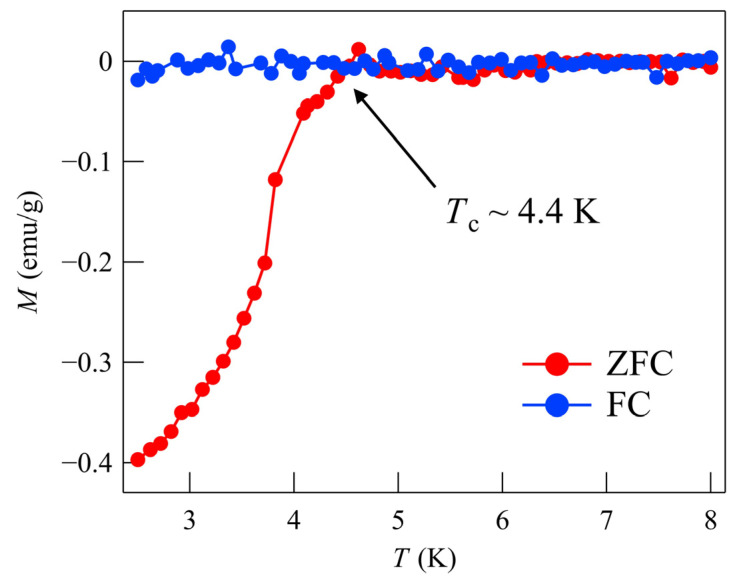
Temperature dependence of the magnetic susceptibility for the In-sample. This measurement was performed on the same crystal used for the resistivity measurements. A magnetic field of 10 Oe was applied along the *c* axis.

The temperature dependence of magnetic susceptibility is shown in Figure 8. A superconducting transition is observed at *T*_c_ ~ 4.4 K. The zero-resistivity temperature, *T*_c_^zero^, is approximately 4.2 K, which is close to the onset temperature of the diamagnetic signal in the *M*(*T*) measurement, approximately 4.4 K. A similar relationship between the zero-resistivity temperature in *ρ*(*T*) and the magnetic onset temperature in *M*(*T*) has also been reported for the ref. compound, (SnSe)_1.16_(NbSe_2_) [18]. Therefore, the *ρ*(*T*) and *M*(*T*) results are reasonably consistent. It is noted that the ZFC signal is not saturated until 2.5 K. It is possible that a lower temperature is required for saturation to be observed, or that the In distribution in the In-sample broadened the transition. Further *M*(*T*) measurements at lower temperatures are needed to confirm this discussion.

The apparent shielding fraction assuming the (SnSe)_2_(NbSe_2_) structure is calculated to be about 320% at 2.5 K. This value exceeds 100% because of the large demagnetization effect arising from the thin platelet-like sample shape. Using the approximation formula proposed by Prozorov and Kogan [30], the demagnetization factor was estimated to be *N* ~ 0.985 for the present measurement geometry. After correcting the apparent shielding fraction using this demagnetization factor, the shielding fraction is estimated to be approximately 77%. The superconducting transition of the In-sample is broader than that of the ref. sample. This broadening may be attributed to a slight inhomogeneity in the In distribution in the In-sample, as the additional phase is not detected by laboratory and synchrotron XRD or TEM measurements.

**Figure 9:**
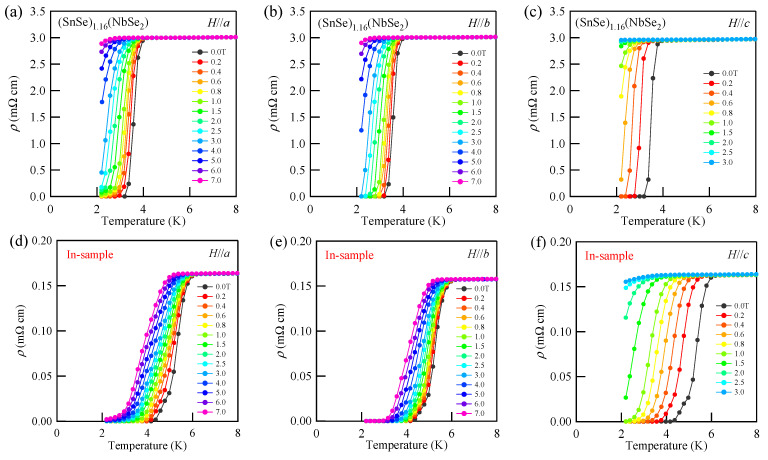
Temperature dependence of electrical resistivity under magnetic fields. Results of the magnetic field along the *a*, *b*, and *c* axes in (SnSe)_1.16_(NbSe_2_) (**a**–**c**) and the In-sample (**d**–**f**).

Figure 9 shows the temperature dependence of the electrical resistivity under magnetic fields for the ref. (a–c) and In-samples (d–f). Superconductivity for both samples is robust to the magnetic field along the in-plane (Figure 9a,b,d,e) compared to that along the *c* axis (Figure 9c,f). This trend originated from the two-dimensional electronic structure due to the layered structure.

**Figure 10:**
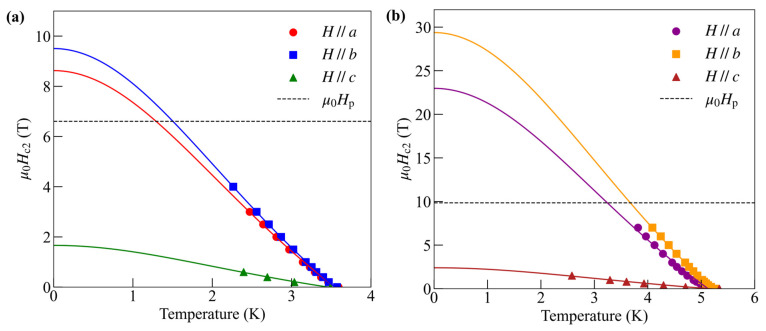
*μ*_0_*H*_c2_-*T*_c_ phase diagram for the ref. sample (**a**) and In-sample (**b**). The dashed lines in both graphs represent the Pauli limit (*μ*_0_*H*_p_), calculated as 1.86*T*_c_.

The temperature dependence of the upper critical field along the *a*, *b*, and *c* axes calculated from Figure 9 is presented in Figure 10. The solid lines in Figure 10 are the fitting lines estimated for an anisotropic two-band superconductor in the Ginzburg–Landau (GL) scenario, expressed as follows [31]:

(1) $$\mu_{0}H_{c2}\left(T\right)={\mu_{0}H}_{c2}\left(0\right)\left[1-{\left(T/T_{c}\right)}^{2}\right]/\left[1+{\left(T/T_{c}\right)}^{2}\right]$$

The reason for adopting the two-band model is that NbSe_2_ has been reported to exhibit multiband superconductivity [32,33,34]. Furthermore, the upper critical fields of NbSe_2_-based misfit superconductors have also been estimated using this model [18,19]. Therefore, this model was also used to estimate the *μ*_0_*H*_c2_ in the In-sample.

In this paper, *μ*_0_*H*_c2_(*T*) values along the *a*, *b* and *c* axes are denoted as *μ*_0_$H_{c2}^{\left|\right|a}$, *μ*_0_$H_{c2}^{\left|\right|b}$ and *μ*_0_$H_{c2}^{\left|\right|c}$, respectively. The upper critical field values at 0 K are estimated from the fitting lines. (SnSe)_1.16_(NbSe_2_) has *μ*_0_$H_{c2}^{\left|\right|a}$(0) = 8.6 T, *μ*_0_$H_{c2}^{\left|\right|b}$(0) = 9.5 T and *μ*_0_$H_{c2}^{\left|\right|c}$(0) = 1.7 T. The In-sample has *μ*_0_$H_{c2}^{\left|\right|a}$(0) = 23.0 T, *μ*_0_$H_{c2}^{\left|\right|b}$(0) = 29.4 T, and *μ*_0_$H_{c2}^{\left|\right|c}$(0) = 2.4 T. The In-sample exhibited higher *μ*_0_*H*_c2_ values than the ref. sample in all directions. The *μ*_0_$H_{c2}^{\left|\right|a}$ and *μ*_0_$H_{c2}^{\left|\right|b}$ of the ref. sample show an analogous temperature dependence. On the other hand, the *μ*_0_$H_{c2}^{\left|\right|b}$ in the In-sample has a higher value in comparison with that of *μ*_0_$H_{c2}^{\left|\right|a}$, indicating the in-plane anisotropy between *μ*_0_$H_{c2}^{\left|\right|a}$ and *μ*_0_$H_{c2}^{\left|\right|b}$ is enhanced slightly in the In-sample. These results demonstrate that the In-sample exhibits higher *T*_c_, *μ*_0_*H*_c2_ and in-plane anisotropy of *μ*_0_*H*_c2_ than the ref. sample. A previous study of (BiSe)_1.10_(NbSe_2_) showed that *μ*_0_$H_{c2}^{\left|\right|b}$ was larger than *μ*_0_$H_{c2}^{\left|\right|a}$ for all temperature ranges, consistent with the current study [22]. However, the in-plane anisotropy of (BiSe)_1.10_(NbSe_2_) is larger than the In-sample. This difference may be attributed to differences in Bi and Sn in the *MX* layers. The origin of this difference is an open question so far.

The value of *μ*_0_*H*_c2_ can be expressed using the anisotropic Ginzburg–Landau relation:

(2) $$\mu_{0}H_{c2}^{i}\left(0\right)=\;\frac{\Phi_{0}}{2\pi \xi_{j}\left(0\right)\xi_{k}\left(0\right)}$$

where *Φ*_0_ is the flux quantum and *ξ* represents the coherence length. The indices of *i*, *j* and *k* represent the cyclic permutation of the directions *a*, *b*, and *c*. The coherence lengths *ξ_i_* (*i* = *a*, *b*, *c*) along each axis were calculated using this equation and the experimental values of *μ*_0_*H*_c2_. All values are summarized in Table 2. The $H_{c2}^{\left|\right|a}(0)$/*H*_p_ and $H_{c2}^{\left|\right|b}(0)$/*H*_p_ of the In-sample are higher than those of the ref. sample. The coherence length along the *c* axis, *ξ_c_*, is significantly smaller than the thickness of the SnSe layers separating the superconducting NbSe_2_ layers in both ref. and In-samples. In particular, the *ξ_c_* of the In-sample is lower than that of the ref. sample. This fact indicates that the two-dimensional electronic structure is enhanced in the In-samples. Since the enhanced two-dimensionality can increase the orbital upper critical field for magnetic fields applied parallel to the layers, the *μ*_0_*H*_p_ restricts the in-plane *μ*_0_*H*_c2_. However, the in-plane *μ*_0_*H*_c2_ in our sample exceeds the *μ*_0_*H*_p_. The NbSe_2_ thin film also shows higher in-plane *μ*_0_*H*_c2_ (~4 *μ*_0_*H*_p_ in trilayers to ~10 *μ*_0_*H*_p_ in a monolayer), which is discussed in Ising pairing in superconductivity [35]. Similar behavior has also been reported in NbSe_2_-based misfit layered compounds. For instance, (PbSe)_1.14_(NbSe_2_)_3_ showed higher in-plane *μ*_0_*H*_c2_ (~4 *μ*_0_*H*_p_). The angular dependence of *μ*_0_*H*_c2_, TDO (tunnel diode oscillator) measurements, and theoretical calculations suggest that Ising superconductivity and the FFLO state are realized, depending on the magnetic field [14]. (LaSe)_1.14_(NbSe_2_)*_n_* (*n* = 1, 2) also has such a higher in-plane *μ*_0_*H*_c2_(~10 *μ*_0_*H*_p_ and ~5 *μ*_0_*H*_p_) [36]. The possibility of Ising superconductivity is discussed based on strong spin–orbit coupling, as confirmed by ARPES, NMR, and first-principles calculations [36,37]. Therefore, the *μ*_0_*H*_c2_ value for exceeding the Pauli paramagnetic limit is suggestive of the existence of the Ising superconductivity. Based on these previous studies related to NbSe_2_ and NbSe_2_-based misfit layered compounds, a similar phenomenon may occur in the Pauli-limit-exceeding in-plane *μ*_0_*H*_c2_ observed in the present In-sample. However, direct experimental evidence for Ising superconductivity, such as angular-dependent *μ*_0_*H*_c2_ measurements or TDO measurements, was not obtained in this study. Therefore, the origin of the large in-plane *μ*_0_*H*_c2_ cannot be determined from the present results alone, and further experimental studies are required.

## 4. Discussion

We discussed the origin of the In-sample properties compared with (SnSe)_1.16_(NbSe_2_). First, we recall the stacking structure of the In-sample. The *c* axis of the In-sample expands, which is due to the thickening of the layer other than NbSe_2_. This expansion is expected to separate the conductive NbSe_2_ layers, thereby enhancing the two-dimensionality of the NbSe_2_ layers. This enhancement results in an increase in *μ*_0_*H*_c2_ along the in-plane directions (*a* and *b* axes), as NbSe_2_ thin films and (LaSe)_1.14_(NbSe_2_)*_n_*. The In-sample also exhibits a higher *T*_c_ than the ref. sample. One possibility leading to a higher *T*_c_ is (1) a change in the number of SnSe layers. However, previous studies on (SnSe)*_m_*(NbSe_2_) have reported that *T*_c_ decreases with increasing *m* [27,38]. Therefore, this scenario cannot account for the observed *T*_c_ enhancement. The other possibility is (2) a change in the carrier density of the NbSe_2_ layer. It has been reported that *T*_c_ decreases with electron doping and increases with hole doping in NbSe_2_ by an ionic liquid gate [39,40]. Furthermore, in misfit layered compounds (*M*Se)(NbSe_2_) (*M* = Sn, Bi, Pb, La), the *M*Se layer has been shown to act as an electron donor to the NbSe_2_ layer, suggesting that the insertion of a SnSe layer may reduce *T*_c_ compared to pristine NbSe_2_ [41,42]. Indeed, the *T*_c_ of (*M*Se)(NbSe_2_) is lower than that of pristine NbSe_2_ [18,20,21,22,23,24]. In addition, the reported relationship between *T*_c_ and the number of *M*Se layers, described by (1), is consistent with this result: *T*_c_ decreases as the number of *M*Se layers increases [27,38]. Furthermore, previous studies on (SnSe)_1.16_(NbSe_2_)*_n_* (*n* = 1, 2) reported that the hole carrier density decreases with increasing the number of SnSe layers, accompanied by a decrease in *T*_c_ [19]. In (LaSe)_1.14_(NbSe_2_)*_n_* (*n* = 1, 2), the *n* = 2 sample has a higher hole carrier density than the *n* = 1 sample [36]. Based on these previous studies, changes in carrier density affect *T*_c_ in (*M*Se)(NbSe_2_). Therefore, the In-sample may also alter the carrier state of the NbSe_2_ layer due to differences in valence between the In and Sn ions, contributing to the change in *T*_c_. This suggestion is consistent with the decrease in resistivity relative to the ref. sample, as shown in Figure 7. However, Hall-effect measurements, carrier concentration analysis, and valence-state measurements have not been performed in the present study. Therefore, the detailed mechanism of the *T*_c_ enhancement cannot be determined from the present results alone. To confirm the suggestion, an evaluation of the carrier concentration and valence of the In ion is needed.

## 5. Conclusions

We investigated the novel sample (In-sample) obtained by the In-addition synthesis in (SnSe)_1.16_(NbSe_2_). In the In-sample, the layer other than the NbSe_2_ layer is thicker, as confirmed by XRD and TEM measurements. On the other hand, the misfit stacking structure was maintained after the In addition. The *T*_c_ of the In-sample increased to 5.4 K in comparison to 3.6 K for the ref. sample. The $H_{c2}^{\left|\right|a}\left(0\right)$/*H*_p_ and $H_{c2}^{\left|\right|b}\left(0\right)$/*H*_p_ were 1.3 and 1.4 for the ref. sample, while they were 2.3 and 3.0 for the In-sample. The calculated *ξ_a_*(0) and *ξ_b_*(0) were almost the same value between the ref. and In-samples. However, the *ξ_c_*(0) in the In-sample is smaller than that of the ref. sample, which is related to the increase in the thickness of the layer other than NbSe_2_. This small *ξ_c_*(0) causes a substantial rise in *μ*_0_$H_{c2}^{\left|\right|a}\left(0\right)$ and *μ*_0_$H_{c2}^{\left|\right|b}\left(0\right)$. Therefore, the enhanced superconducting properties of the In-sample are likely associated with both In incorporation and the accompanying structural modification. These results are essential for controlling the novel superconducting state in misfit layered compounds with higher *μ*_0_*H*_c2_.

## Figures and Tables

**Table 1:** Summary of the lattice parameters for the ref. sample and the In-sample. The lattice parameters for the In-sample were independently calculated from the SAED patterns and from the single-crystal X-ray diffraction (SC-XRD) measurements. For the ref. sample, the lattice parameters were taken from powder XRD, as reported in previous work [18].

In-Sample					(SnSe)_1.16_(NbSe_2_) [18]			
Subsystem	*a* (Å)	*b* (Å)	*c* (Å)	method	*a* (Å)	*b* (Å)	*c* (Å)	method
Square	5.76(8)	5.88(6)	14.8(1)	SAED	5.940	5.968	12.340	P-XRD
Triangular	3.38(4)	5.88(6)			3.444	5.968	24.68	
Square	5.628	5.935	14.398	SC-XRD				
Triangular	3.384	5.919						

**Table 2:** Lattice constants along the *c* axis, *T*_c_, *μ*_0_*H*_c2_(0) for each axis, *H*_c2_/*H*_p_, and coherence lengths for each sample.

	(SnSe)_1.16_(NbSe_2_)	In-Sample
*c*	12.32	14.77
*T*_c_	3.6	5.4
*μ*_0_$H_{c2}^{\left|\right|a}$(0)	8.6	23.0
*μ*_0_$H_{c2}^{\left|\right|b}$(0)	9.5	29.4
*μ*_0_$H_{c2}^{\left|\right|c}$(0)	1.7	2.4
$H_{c2}^{\left|\right|a}$(0)/*H*_p_	1.3	2.3
$H_{c2}^{\left|\right|b}$(0)/*H*_p_	1.4	3.0
$H_{c2}^{\left|\right|c}$(0)/*H*_p_	0.3	0.2
*ξ_a_* (Å)	13.4	10.3
*ξ_b_* (Å)	14.8	13.2
*ξ_c_* (Å)	2.6	1.1
*γ* *_ab_*	1.1	1.3
*γ* *_ac_*	5.2	9.6
*γ* *_bc_*	5.7	12

## Data Availability

The original contributions presented in this study are included in the article. Further inquiries can be directed to the corresponding author.

## References

[1] Ando F., Miyasaka Y., Li T., Ishizuka J., Arakawa T., Shiota Y., Moriyama T., Yanase Y., Ono T. (2020). Observation of superconducting diode effect. Nature.

[2] Smidman M., Salamon M.B., Yuan H.Q., Agterberg D.F. (2017). Superconductivity and spin–orbit coupling in non-centrosymmetric materials: A review. Rep. Prog. Phys..

[3] Fischer M.H., Sigrist M., Agterberg D.F., Yanase Y. (2023). Superconductivity and Local Inversion-Symmetry Breaking. Annu. Rev. Condens. Matter Phys..

[4] Ng N., McQueen T.M. (2022). Misfit layered compounds: Unique, tunable heterostructured materials with untapped properties. APL Mater..

[5] Meerschaut A. (1996). Misfit layer compounds. Curr. Opin. Solid State Mater. Sci..

[6] Aliev S.B., Tenne R. (2020). Quaternary Misfit Compounds—A Concise Review. Crystals.

[7] Lorenz T., Joswig J.-O., Seifert G. (2014). Two-dimensional and tubular structures of misfit compounds: Structural and electronic properties. Beilstein J. Nanotechnol..

[8] Khadiev A., Sreedhara M.B., Hettler S., Novikov D., Arenal R., Tenne R. (2024). Misfit Layered Compounds: Insights into Chemical, Kinetic, and Thermodynamic Stability of Nanophases. Acc. Chem. Res..

[9] Wiegers G.A. (1993). Misfit Layer Compounds Based on Double Layers MX and Sandwiches TX_2_ (M = Sn, Pb, Sb, Bi, Ln; T = Ti, V, Cr, Nb, Ta; X = S, Se). Jpn. J. Appl. Phys..

[10] Wiegers G.A. (1996). Misfit layer compounds: Structures and physical properties. Prog. Solid State Chem..

[11] Sankar R., Peramaiyan G., Muthuselvam I.P., Wen C.-Y., Xu X., Chou F.C. (2018). Superconductivity in a Misfit Layered (SnS)_1.15_(TaS_2_) Compound. Chem. Mater..

[12] Giang N., Xu Q., Hor Y.S., Williams A.J., Dutton S.E., Zandbergen H.W., Cava R.J. (2010). Superconductivity at 2.3 K in the misfit compound (PbSe)_1.16_(TiSe_2_)_2_. Phys. Rev. B.

[13] Nader A., Lafond A., Briggs A., Meerschaut A., Roesky R. (1998). Structural characterization and superconductivity in the misfit layer compound (LaSe)_1.14_(NbSe_2_). Synth. Met..

[14] Itahashi Y.M., Nohara Y., Chazono M., Matsuoka H., Arioka K., Nomoto T., Kohama Y., Yanase Y., Iwasa Y., Kobayashi K. (2025). Misfit layered superconductor (PbSe)_1.14_(NbSe_2_)_3_ with possible layer-selective FFLO state. Nat. Commun..

[15] Agarwal T., Patra C., Manna P., Srivastava S., Mishra P., Sharma S., Singh R.P. (2025). Anomalous magnetotransport in the superconducting architecturally misfit layered system (PbS)_1.13_TaS_2_. Phys. Rev. B.

[16] Grosse C., Alemayehu M.B., Falmbigl M., Mogilatenko A., Chiatti O., Johnson D.C., Fischer S.F. (2016). Superconducting ferecrystals: Turbostratically disordered atomic-scale layered (PbSe)_1.14_(NbSe_2_)_n_ thin films. Sci. Rep..

[17] Wiegers G.A., Zhou W.Y. (1991). The misfit layer compound (SnSe)_1.16_NbSe_2_. Mat. Res. Bull..

[18] Bai H., Yang X., Liu Y., Zhang M., Wang M., Li Y., Ma J., Tao Q., Xie Y., Cao G.-H. (2018). Superconductivity in a misfit layered compound (SnSe)_1.16_(NbSe_2_). J. Phys. Condens. Matter.

[19] Bai H., Qiao L., Li M., Ma J., Yang X., Li Y., Tao Q., Xu Z.-A. (2020). Multi-band Superconductivity in a misfit layered compound (SnSe)_1.16_(NbSe_2_)_2_. Mater. Res. Express.

[20] Samuely P., Szabó P., Kacmarcik J., Meerschaut A., Cario L., Jansen A.G.M., Cren T., Kuzmiak M., Šofranko O., Samuely T. (2021). Extreme in-plane upper critical magnetic fields of heavily doped quasi-two-dimensional transition metal dichalcogenides. Phys. Rev. B.

[21] Zhong H., Zhang H., Zhang H., Bao T., Zhang K., Xu S., Luo L., Rousuli A., Yao W., Denlinger J.D. (2023). Revealing the two-dimensional electronic structure and anisotropic superconductivity in a natural van der Waals superlattice (PbSe)_1.14_NbSe_2_. Phys. Rev. Mater..

[22] Matsuzawa S., Kitano H., Pyon S., Tamegai T. (2023). Two-dimensional Superconductivity in Misfit Layered Compound (BiSe)_1.10_NbSe_2_. J. Phys. Conf. Ser..

[23] Matsuzawa S., Pyon S., Tamegai T. (2022). Characterizations of Anisotropic Superconductivity in (BiSe)_1+δ_NbSe_2_. J. Phys. Conf. Ser..

[24] Zehetmayer M., Weber H.W. (2010). Experimental evidence for a two-band superconducting state of NbSe_2_ single crystals. Phys. Rev. B.

[25] Gómez-Herrero A., Landa-Cánovas A.R., Otero-Díaz L.C. (2015). TEM study of the (SbS)_1+δ_(NbS_2_)_n_, (n=1, 2, 3; δ~1.14, 1.20) misfit layer phases. J. Solid State Chem..

[26] Alemayehu M.B., Falmbigl M., Ta K., Johnson D.C. (2015). Effect of Local Structure of NbSe_2_ on the Transport Properties of ([SnSe]_1.16_)_1_(NbSe_2_)_n_ Ferecrystals. Chem. Mater..

[27] Shu R., Nagao M., Yamamoto C., Yamanaka J., Maruyama Y., Watauchi S., Tanaka I. (2024). Growth and characterization of superconducting bulk crystal [(SnSe)_1+δ_]_m_(NbSe_2_) misfit layer compounds. J. Alloys Compd..

[28] Niu R., Li J., Zhen W., Xu F., Weng S., Yue Z., Meng X., Xia J., Hao N., Zhang C. (2024). Enhanced Superconductivity and Critical Current Density Due to the Interaction of InSe_2_ Bonded Layer in (InSe_2_)_0.12_NbSe_2_. J. Am. Chem. Soc..

[29] Liu H., Li Y., Ge J., Liu Y., Liu S., Wang H., Xing Y. (2025). Transport properties of (InSe_2_)_*x*_NbSe_2_ with enhanced *T*_*c*_ and *H*_*c*2_. Phys. Rev. B.

[30] Prozorov R., Kogan V.G. (2018). Effective Demagnetizing Factors of Diamagnetic Samples of Various Shapes. Phys. Rev. Appl..

[31] Changjan A., Udomsamuthirun P. (2011). The critical magnetic field of anisotropic two-band magnetic superconductors. Solid State Commun..

[32] Yokoya T., Kiss T., Chainani A., Shin S., Nohara M., Takagi H. (2001). Fermi Surface Sheet-Dependent Superconductivity in 2H-NbSe_2_. Science.

[33] Boaknin E., Tanatar M.A., Paglione J., Hawthorn D., Ronning F., Hill R.W., Sutherland M., Taillefer L., Sonier J., Hayden S. (2003). Heat Conduction in the Vortex State of NbSe_2_: Evidence for Multiband Superconductivity. Phys. Rev. Lett..

[34] Huang C.L., Lin J.-Y., Chang Y.T., Sun C.P., Shen H.Y., Chou C.C., Berger H., Lee T.K., Yang H.D. (2007). Experimental evidence for a two-gap structure of superconducting NbSe_2_: A specific-heat study in external magnetic fields. Phys. Rev. B.

[35] Xi X., Wang Z., Zhao W., Park J.-H., Law K.T., Berger H., Forró L., Shan J., Mak K.F. (2016). Ising pairing in superconducting NbSe_2_ atomic layers. Nat. Phys..

[36] Samuely T., Wickramaratne D., Gmitra M., Jaouen T., Šofranko O., Volavka D., Kuzmiak M., Haniš J., Szabó P. (2023). Protection of Ising spin-orbit coupling in bulk misfit superconductors. Phys. Rev. B.

[37] Shan M., Li S., Yang Y., Zhao D., Li J., Nie L., Wu Z., Zhou Y., Zheng L., Kang B. (2024). Anisotropic Spin Fluctuations Induced by Spin-Orbit Coupling in a Misfit Layer Compound (LaSe)_1.14_(NbSe_2_). Adv. Sci..

[38] Alemayehu M.B., Falmbigl M., Ta K., Grosse C., Westover R.D., Bauers S.R., Fischer S.F., Johnson D.C. (2015). Structural and Electrical Properties of ([SnSe]_1+δ_)_m_(NbSe_2_)_1_ Compounds: Single NbSe_2_ Layers Separated by Increasing Thickness of SnSe. Chem. Mater..

[39] Xi X., Berger H., Forró L., Shan J., Mak K.F. (2016). Gate Tuning of Electronic Phase Transitions in Two-Dimensional NbSe_2_. Phys. Rev. Lett..

[40] Yoshida M., Ye J., Nishizaki T., Kobayashi N., Iwasa Y. (2016). Electrostatic and electrochemical tuning of superconductivity in two-dimensional NbSe_2_ crystals. Appl. Phys. Lett..

[41] Leriche R.T., Palacio-Morales A., Campetella M., Tresca C., Sasaki S., Brun C., Debontridder F., David P., Arfaoui I., Šofranko O. (2021). Misfit Layer Compounds: A Platform for Heavily Doped 2D Transition Metal Dichalcogenides. Adv. Funct. Mater..

[42] Zullo L., Marini G., Cren T., Calandra M. (2023). Misfit Layer Compounds as Ultratunable Field Effect Transistors: From Charge Transfer Control to Emergent Superconductivity. Nano Lett..

